# Books and Pamphlets Received

**Published:** 1884-03-20

**Authors:** 


### *BOOKS AND PAMPHLETS RECEIVED.*

*The Popular Science Monthly*, conducted by E. L. & W. J. Youmans, and published by D Appleton & Co., New York, at $5.00 per annum.

This Journal is now justly regarded as the best and ablest exponent of the advanced thought of the age, especially in referenc to scientific subjects. It contains 143 large octavo pages, neatly designed and typographically excellent. It is a journal largely read by advanced thinkers and those having a taste for scientific subjects, and especially well suited to investigating and progressive minds in the medical Profession.

*Medical Directory of Philadelphia*, edited by Samuel B. Hoppin, M.D. Philadelphia. P. Blakiston, Son & Co., 1012 Walnut St., 1884.

The Directory is replete with much useful information, being a complete Directory of all the physicians, the societies, hospitals, homes, charities, etc. Not the least valuable of the information it contains is the interesting list of medical publications, etc., by the Publishing House of P. Blakiston, Son & Co., 1012 Walnut St., Philadelphia.

